# Rapid detection of β-lactamase activity using the rapid Amp NP test

**DOI:** 10.1128/spectrum.00782-24

**Published:** 2025-03-06

**Authors:** Patrice Nordmann, Nicolas Helsens, Nicolas Kieffer, Camille Tinguely, Gilbert Greub, Laurent Poirel

**Affiliations:** 1 European Institute for Emerging Antibiotic Resistance, University of Fribourg, Fribourg, Switzerland; 2 Medical and Molecular Microbiology, Faculty of Science and Medicine, University of Fribourg, Fribourg, Switzerland; 3 Swiss National Reference Center for Emerging Antibiotic Resistance (NARA), University of Fribourg, Fribourg, Switzerland; 4 Institute for Microbiology, University Hospital and University of Lausanne, Lausanne, Switzerland; 5 Clinical Microbiology Unit, Pasteur Institute of Lille, Lille, France; Universidade de Sao Paulo, São Paulo, Brazil

**Keywords:** β-lactamase, UTI, rapid diagnostic test, Gram negative

## Abstract

Urinary tract infections (UTIs) are the most common bacterial infections in humans. They are mainly caused by *Escherichia coli* and other Enterobacterales for which increasing resistance to antibiotics and in particular to β-lactams is extensively reported. The detection of β-lactam resistance phenotypes is currently time-consuming (18 h). Hence, most treatments are given without any results of antibiotic susceptibility testing and may involve broad-spectrum antibiotics. A biochemical diagnostic test has been developed to rapidly evaluate the production of β-lactamases (and consequently the β-lactam resistance) from cultures (10^4^ and 10^5^ CFU/mL) of Gram-positive and Gram-negative isolates representative of bacterial species as a source of UTIs (*n* = 112). It relies on a centrifugation step after a 90 min preliminary culture and the detection of β-lactamase activity with nitrocefin as substrate using a special filter. Overall, the test gave a positive result for 87.6% of the tested resistant strains with a bacterial load of 10^5^ CFU/mL, and a positive result for 100% of tested extended-spectrum β-lactamases and for carbapenemase producers at the same load. This cost-effective test can be performed in any laboratory and ultimately shall be tested at the patient side and at the general practitioner. Its turn-around-time to get results is less than 2 h. After further improvements, the results obtained with this proof-of-concept test suggest that its use may contribute to rapidly guide the treatment of non-complicated UTIs and, therefore, limit the use of broad-spectrum antibiotics and the emergence of antibiotic resistance.

**IMPORTANCE:**

This work reports on a totally novel diagnostic technique, the Rapid Amp NP test for the identification of amoxicillin/ampicillin resistance in bacteria that are sources of non-complicated urinary tract infections. Those preliminary results obtained with cultured bacteria are promising. We believe its future use may contribute to reconsider aminopenicillins as a first line therapy for treating UTI infections. The corresponding patent of this test obtained both for the United States and Europe may contribute to its further industrialization.

## INTRODUCTION

An estimated 700,000 deaths can be attributed every year globally to antibiotic-resistant bacteria, and this number is projected to increase to 10 million deaths every year by 2050, coming along with a heavy economic burden (1). Furthermore, pharmaceutical research and development for antibiotic molecules account for a small fraction of the total research investment, and less and less new molecules are put on the market every year (1, 2). It becomes, therefore, urgent to reduce the use of antibiotics globally, especially those of critical clinical significance and of broad-spectrum activity. For this purpose, the implementation of rapid and easy-to-use diagnostic tests to identify and characterize resistant bacteria in clinical samples is of primary importance to optimize antibiotic stewardship.

Enterobacterales account for the most significant part of community-acquired infections, many of which express resistance to β-lactam antibiotics (3, 4), due to the production of narrow- to broad-spectrum-β-lactamases (4–6). In Europe, resistance to aminopenicillins has been observed in 57.4% of *Escherichia coli* isolates, resistance to expanded-spectrum cephalosporins in 15.1% of *E. coli* isolates (7), and resistance to carbapenems being much lower, its rates varying from virtually 0% to 7%, in *E. coli*, depending on the country (8). Resistance to aminopenicillins in Enterobacterales is due to β-lactamases such as SHV/LEN enzymes in *Klebsiella pneumoniae*, AmpC enzymes in *Enterobacter*, *Serratia* sp., *Morganella* sp., or by acquired narrow-spectrum enzymes such as TEM-1, TEM-2, SHV-1 in many *Enterobacterales* species such as *E. coli*. Of note, clinically significant resistance to penicillins in *E. coli* involves at least the expression of a β-lactamase, meaning that lack of β-lactamase production can be safely translated in full β-lactam susceptibility in that species (9, 10).

Urinary tract infections (UTIs) are the most prevalent bacteria-related infectious diseases in humans, with an estimated age-standardized incidence rate of 44.5/1,000 persons per year in Europe in 2019 (11). More than 80% of UTIs are caused by *E. coli,* and other notable causative agents are *K. pneumoniae*, *Enterococcus faecalis*, *Staphylococcus saprophyticus*, *Pseudomonas aeruginosa*, *Proteus mirabilis,* and *Staphylococcus aureus* (12). In addition, reports of multidrug-resistant pathogens causing UTIs are multiplying worldwide (13–16), and the emphasis can be put on extended-spectrum β-lactamases (ESBL)- and carbapenemase-producing Enterobacterales (17). Hence, the empirical treatment of cystitis (non-complicated UTI) currently involves broad-spectrum antibiotics such as fosfomycin, nitrofurantoin, or fluoroquinolones (ciprofloxacin or ofloxacin) (18, 19). Noteworthy, empirical use of penicillins for treating UTIs is prevented by the relatively high rate of β-lactamase-producing *E. coli*, as mentioned above. Nevertheless, if considering this rate to be ca. 50% in many countries (for instance in Europe), it means that ca. 50% of *E. coli* strains responsible for UTIs do not produce any β-lactamase and, therefore remain susceptible to penicillins.

The rapid detection of resistance genes in bacteria responsible for infections is critical to limit their dissemination and to adapt the first-line antibiotic treatment in response. Among the different diagnostic tools available, there are (i) phenotypic techniques, in which case the β-lactam resistance patterns are looked for and (ii) molecular techniques, in which case the resistance gene itself is detected. Those different techniques may often lack specificity or sensitivity, are time-consuming or costly, therefore preventing them from a routine and large-scale application. Several rapid tests have been developed, involving the direct detection of specific β-lactamase activity directly from cultured bacteria thanks to the chromogenic properties of different reagents, such as those used with Rapid ESBL NP test (17, 20), the Carba NP test (21) or one of its derivative, and the NitroSpeed Carba NP test (22). They provide fast, sensitive, and specific results for the detection of either ESBLs or carbapenemases in bacterial samples. They require a preliminary 16–24 h growth step in order to obtain cultures for high level of bacterial concentration. The present study proposes a novel test, namely, the Rapid Amp NP test. The ultimate goal is to detect β-lactamase activity from urine samples. It relies on the detection of any kind of β-lactamase activity (narrow or extended-spectrum activity) based on the chromogenic properties of nitrocefin. The proof-of-concept of the test was developed here with culture of bacteria containing bacterial loads comparable to those of non-complicated UTI samples and allows this detection in a short time period (2–3 h).

## MATERIALS AND METHODS

### Strain selection

A collection of 112 bacterial isolates recovered from clinical samples (mostly urine, blood culture, or rectal swabs) and from various countries was used for this study. All strains were tested for their β-lactam resistance phenotypes, and β-lactamase-encoding genes were characterized using a PCR approach followed by DNA sequencing for the surveillance activity of the Swiss National Reference Center for Emerging Antibiotic Resistance. They were selected to represent the diversity of bacterial species responsible for UTI (23), namely, *E. coli*, *Acinetobacter baumannii*, *Citrobacter freundii*, *Enterobacter cloacae*, *Enterococcus faecalis*, *Enterococcus faecium*, *Hafnia alvei*, *Klebsiella oxytoca*, *K. pneumoniae*, *P. mirabilis*, *P. aeruginosa*, and *S. aureus*. The collection included 97 β-lactamase-producing isolates producing either Ambler class A β-lactamases (CTX-M, IMI-1, KPC, SHV, TEM, and VEB), class B (IMP, NDM, and VIM), class C (natural AmpC, CMY-2, and DHA-1), or class D (OXA), as well as three methicillin-resistant that are β-lactamase-positive *S. aureus* (MRSA). It also included 15 β-lactam-susceptible and β-lactamase-negative strains, as negative controls. Susceptibility testing of the selected isolates for ampicillin, amoxicillin-clavulanic acid, ticarcillin piperacillin, and cefazolin was assessed using the broth micro-dilution method and interpreted according to the latest EUCAST guidelines (https://www.eucast.org/clinical_breakpoints). The selected strains along with their resistance determinants and phenotypic resistance profiles are listed in Table 1.

**TABLE 1:** Features of the tested strains, results of the Rapid Amp NP detection time according to the inoculum size, of the cefinase test performed on cultures of bacteria grown on agar plates and minimal inhibitory concentrations of several β-lactams

			Rapid Amp NP detection time (min)					Minimal inhibitory concentration (µg/mL)				
β-Lactamase (*n*^*a*^)	Species	β-Lactamase (*n*)	10^5^ CFU/mL	10^4^ CFU/mL	10^3^ CFU/mL	10^2^ CFU/mL	Cefinase test	AMP^*b*^	AMC	TIC	PIL	CZN
Non-β-lactamase producers (15)	*Enterococcus faecalis*	Wild type (1)	−^*c*^	−	−	−	−	≤ 0.125	0.25	32	1	8
	*Staphylococcus aureus*	Wild type (1)	−	−	−	−	−	≤0.125	≤0.125	1	0.5	0.125
Non-ESBL producers (39)	*Escherichia coli*	Wild type (12)	−	−	−	−	−	1 to 2	≤1 to 16	1 to 8	≤0.125 to 1	0.25 to 8
	*Proteus mirabilis*	Wild type (1)	−	−	−	−	−	>128	2	32	16	0.25
					−	−						
	*Enterococcus faecalis*	Penicillinase (1)	−	−	−	−	^*d*^+	≤0.25	≤0.25	16	1	2
	*Enterococcus faecium*	Penicillinase (1)	5	5	30	−	+	>64	>128	>128	> 128	>128
	*Staphylococcus aureus*	MRSA^*e*^ (3)	−	−	−	−	+	16 to 64	4 to 16	32 to 128	64 to >128	16 to > 128
	*Acinetobacter baumannii*	Overproduced AmpC (1)	5	−	−	−	+	64	64	64	32	> 128
	*Citrobacter freundii*	Overproduced AmpC (1)	5	30	−	−	+	>128	>128	>128	> 128	> 128
	*Enterobacter cloacae*	Overproduced AmpC (2)	−	−	−	−	+	8 to >128	8 to >128	1 to 64	1 to 16	> 128
	*Escherichia coli*	CMY-2 (1)	5	−	−	−	+	>128	>128	>128	> 128	> 128
		DHA-1 (1)	30	−	−	−	+	>128	>128	>128	>128	> 128
		OXA-1 (3)	5 (1); −(2)	−	−	−	+	>128	>128	>128	64 to >128	2 to > 128
		SHV-1 (2)	5	10 to 120	120 (1); −(1)	−	+	>128	16	>128	>128	32 to > 128
		TEM-1 (10)	5 to 30	5 (2); 30 (1); 60 (2); − (5)	20 (1); 60 (1); − (8)	−	+	>128	2 to >128	>128	> 128	2 to > 128
		Overproduced AmpC (2)	10 (1); −(1)	−	−	−	+	8 to >128	8 to 32	8 to >128	2 to >128	2 to 16
	*Hafnia alvei*	Overproduced AmpC (1)	5	20	−	−	+	>128	>128	32	32	> 128
	*Klebsiella oxytoca*	Natural penicillinase (2)	5	5 to 10	60 (1); −(1)	−	+	>128	2 to >128	>128	>128	> 128
	*Klebsiella pneumoniae*	Natural penicillinase (4)	20 (1); 120 (1); − (2)	60 (1); − (3)	−	−	+	16 to >128	1 to 8	16 to 128	2 to >128	0.25 to 4
	*Proteus mirabilis*	CMY-2 (1)	5	10	−	-	+	> 128	64	> 128	128	> 128
		Penicillinase (1)	60	−	−	−	+	>128	1	64	8	32
	*Pseudomonas aeruginosa*	Overproduced AmpC (2)	5 (1); − (1)	−	−	−	+	>128	>128	128	16 to 128	> 128
					−	−						
ESBL producers (22)	*Enterobacter cloacae*	TEM-101 (1)	5	5	−	−	+	>128	>128	>128	>128	> 128
	*Escherichia coli*	CTX-M-1 + TEM-1 (1)	5	30	−	−	+	>128	16	>128	>128	> 128
		CTX-M-1 (2)	5	5 to 60	−	−	+	>128	16	>128	>128	> 128
		CTX-M-14 + TEM-1 (1)	20	−	−	−	+	>128	16	>128	>128	> 128
		CTX-M-14 (1)	5	−	−	−	+	>128	16	>128	>128	> 128
		CTX-M-15 (3)	5	20 (2); − (1)	−	−	+	>128	16 to >128	>128	>128	> 128
		CTX-M-2 (1)	5	20	−	−	+	>128	16	>128	>128	> 128
		CTX-M-32 (1)	60	−	−	−	+	>128	2	>128	128 to > 128	> 128
		CTX-M-8 (1)	5	20	−	−	+	>128	>128	>128	>128	> 128
		CTX-M-9 (1)	5	−	−	−	+	>128	>128	>128	>128	> 128
		SHV-12 + TEM-1 (1)	5	120	−	−	+	>128	4	>128	>128	64
		SHV-12 (2)	5 to 10	−	−	−	+	>128	2 to 4	>128	>128	> 128
		TEM-12 (1)	20	−	−	−	+	>128	32	>128	>128	64
		TEM-52 (1)	10	120	−	−	+	>128	4	>128	>128	> 128
		VEB-1 (2)	5	20 (1); − (1)	−	−	+	>128	16 to <128	>128	>128	64
	*Proteus mirabilis*	TEM-21 (1)	10	−	−	−	+	>128	1	>128	>128	> 128
		VEB-1 (1)	10	60	−	−	+	>128	64	>128	>128	> 128
					−	−						
Carbapenemase producers (36)	*Enterobacter cloacae*	IMI-1 (1)	5	−	−	−	+	>128	>128	64	4	> 128
	*Escherichia coli*	IMP-1 (1)	10	−	−	−	+	>128	>128	>128	>128	> 128
		IMP-8 (1)	20	120	−	−	+	>128	>128	>128	>128	> 128
		KPC-2 + TEM-1; OXA-1 (1)	5	20	60	−	+	>128	>128	>128	>128	> 128
		KPC-2 (2)	5	20 to 60	−	−	+	>128	>128	64 to >128	64 to >128	> 128
		KPC-3 (2)	5	5 to 30	30 (1); − (1)	−	+	>128	>128	>128	> 128	> 128
		NDM-1 + CMY-6; CTX-M-15; OXA-1; TEM-1B (1)	5	5	60	−	+	>128	>128	>128	> 128	> 128
		NDM-1 + OXA-1; OXA-2; CTX-M-15; TEM-1 (1)	5	5	60	−	+	>128	>128	>128	>128	> 128
		NDM-1 + CMY-16; OXA-10; CTX-M-15 (1)	10	60	−	−	+	>128	>128	>128	64	> 128
		NDM-1 (2)	5 to 30	20 to 120	−	−	+	>128	>128	>128	>128	> 128
		NDM-19 + TEM-1(1)	20	60	120	−	+	>128	>128	>128	>128	> 128
		NDM-4 + TEM-1; CTX-M-15; CMY-2; OXA-1 (1)	5	30	−	−	+	>128	>128	>128	>128	> 128
		NDM-5 +CMY (1)	5	60	−	−	+	>128	>128	>128	>128	> 128
		NDM-5 + CTX M-24; TEM-1 (1)	5	10	−	−	+	>128	>128	>128	>128	> 128
		NDM-5 (1)	5	30	−	−	+	>128	>128	>128	>128	> 128
		NDM-7 + TEM-1 (1)	30	−	−	−	+	>128	>128	>128	>128	> 128
		OXA-181 + CTX M-1 (1)	5	10	30	−	+	>128	>128	>128	>128	> 128
		OXA-181 + CTX M-27 (1)	10	−	−	−	+	>128	>128	>128	>128	> 128
		OXA-181 (1)	20	60	−	−	+	>128	>128	>128	>128	16
		OXA-244 + CTX M-27 (1)	5	10	−	−	+	>128	>128	>128	>128	> 128
		OXA-48 (2)	5	10 to 20	−	−	+	>128	>128	>128	>128	> 128
		OXA-484 + CTX M-15 (1)	5	10	−	−	+	>128	>128	>128	> 128	> 128
		VIM-1 (1)	10	60	−	−	+	>128	>128	>128	>128	> 128
		VIM-4 (1)	5	20	−	−	+	>128	>128	>128	>128	> 128
	*Klebsiella oxytoca*	KPC-3 (1)	5	5	120	−	+	>128	>128	>128	>128	> 128
		NDM-1 (1)	5	−	−	−	+	>128	>128	>128	>128	> 128
	*Klebsiella pneumoniae*	KPC-2 (3)	5 to 20	20 to 120	−	−	+	>128	>128	>128	>128	> 128
		OXA-204 (1)	10	−	−	−	+	>128	>128	>128	>128	> 128
		VIM-1 (1)	5	30	−	−	+	>128	>128	>128	>128	> 128
	*Pseudomonas aeruginosa*	IMP-7 (1)	60	−	−	−	+	>128	>128	>128	>128	> 128

^*a*^ *n*, number of isolates.

^*b*^ AMP, ampicillin; AMC, amoxicillin-clavulanic acid; TIC, ticarcillin; PIL, piperacillin; CZN, cefazolin.

^*c*^ No detection.

^*d*^ Positive result of the cefinase test.

^*e*^ MRSA, methicillin-resistant *Staphylococcus aureus*.

### Detection of β-lactamase

**Fig 1:**
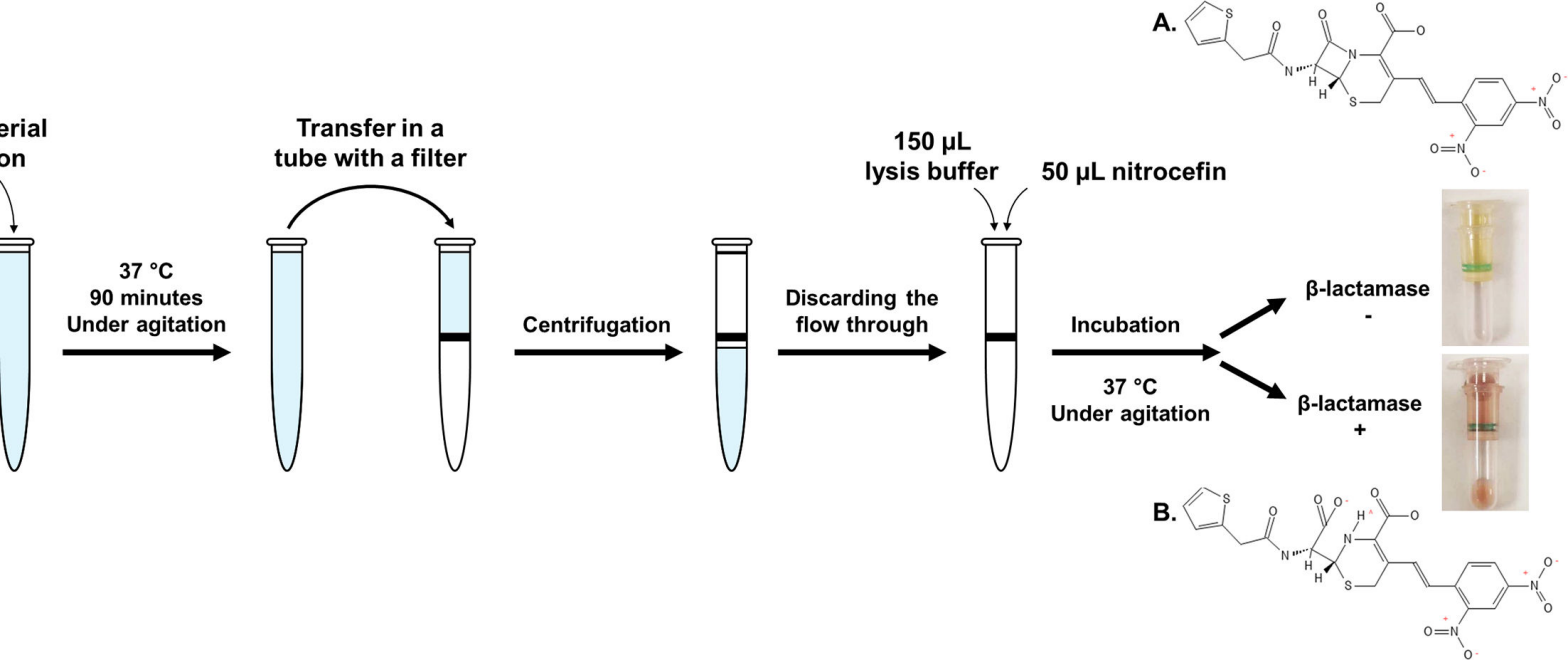
Principle of the Rapid Amp NP test for detection of the β-lactamase activity from cultured bacteria. The hydrolysis of the nitrocefin resulted in the opening of its β-lactam ring and change of its color from (A) yellow in its native form to (B) red in its hydrolyzed form.

In order to assess the ability of the test to detect the occurrence of β-lactamase-producing bacteria at different bacterial loads, broth cultures of the strains were inoculated at an initial inoculum of 10^5^ CFU/mL and 10^4^ CFU/mL, considering that the threshold for the diagnosis of regular UTI is commonly admitted be around 10^5^ CFU/mL (17, 24). Strains were previously cultured at 37°C on UriSelect 4 agar (Bio-Rad, Marnes-la-Coquette, France) for 16–20 h. The bacterial cultures were then grown overnight in LB broth (Carl Roth, Lille, France) and diluted in 10 mL distilled water supplemented with 0.85% NaCl in order to obtain a 0.5 McFarland density, controlled by a densitometer but also by plate colony counting on LB agar medium. A serial 10-fold dilution was then performed in LB broth, resulting in 10 mL bacterial suspensions at 10^2^ to 10^5^ CFU/mL. The resulting suspension was incubated at 37°C under agitation for 90 min and then filtered through a 0.2 µm nylon membrane (GVS Centrex Centrifuge Filter, GVS, Sanford, USA) using a 2-min centrifugation step at 3,200 x *g* (Fig. 1). After discarding the liquid culture medium as a result of the centrifugation step, 150 µL B-PER II (Thermo Scientific Pierce, Rockford, IL, USA) was added to the top surface of the filter in order to lyse the bacterial cells, followed by 50 µL nitrocefin (TRC, Toronto, Canada) at 1 mg/mL. Nitrocefin is a chromogenic cephalosporin which is yellow in its native form and turns red upon hydrolysis by a β-lactamase (25). The filters were then incubated at 37°C for an additional 2 h (maximum) step under shaking at 150 oscillations per minutes. The results were checked regularly at 5, 10, 20-, 30-, 60-, and 120 min endpoints.

Each isolate was also checked for β-lactamase activity by the same nitrocefin component directly from cultured bacteria on plates. A 10 µL loop-full of bacterial colonies was suspended in 200 µL B-PER II, and then 50 µL nitrocefin (1 mg/mL) was added to the suspension. Results were read within 5 min.

Other chromogenic reactions, such as the hydrolysis of the chromogenic cephalosporin CENTA or the identification of pH change caused by β-lactamase hydrolysis or modification of the hydrolysis buffer, were also tested for the detection of β-lactamase activity. None of them gave better results than nitrocefin and the B-PER II buffer (data not shown).

## RESULTS AND DISCUSSION

The Rapid Amp NP test relies on the chromogenic properties of nitrocefin for the detection of any β-lactamase activity. Most β-lactamase (and consequently at least aminopenicillin resistance) detection tests are performed on bacterial colonies, after a bacterial growth taking ca. 18 h. Instead, the Rapid Amp NP test could be applied directly on broth cultures after a 90 min incubation period. It has an overall processing time of ca. 2 h.

The Rapid Amp NP gave true positive results with 87.6% of the tested β-lactam-resistant strains, when the starting bacterial inoculum was 10^5^ CFU/mL (Table 2). At this concentration, a positive result was obtained for 69.2% of the tested strains producing β-lactamases with the exclusion of ESBL or carbapenemases producers (Table 2). As previously described, this relatively poor detection rate could be explained by a weak hydrolytic activity toward nitrocefin for several type β-lactamases such as cephalosporinases (26), a low amount of β-lactamase produced due to a reduced β-lactamase gene copy number, or to a low level of expression of those genes (22).

**TABLE 2:** Interpretation of the results obtained with RapidAmp NP test according to the type of β-lactamase produced, compared to the results expected considering the β-lactam resistance phenotypes of the tested strains^*a*^

Type of produced β-lactamases	Tested concentrations		Expected results	
			R	S
Non-β-lactamase producers (*n* = 15)	**10^5^ CFU/mL**	**R**	–^*b*^	0
		**S**	–	15 (100 %)
	**10^4^ CFU/mL**	**R**	–	0
		**S**	–	15 (100 %)
Non-ESBL producers (*n* = 39)	**10^5^ CFU/mL**	**R**	27 (69.2 %)	–
		**S**	12 (30.8 %)	–
	**10^4^ CFU/mL**	**R**	14 (35.9 %)	–
		**S**	25 (64.1 %)	–
ESBL producers (*n* = 22)	**10^5^ CFU/mL**	**R**	22 (100%)	–
		**S**	0	–
	**10^4^ CFU/mL**	**R**	12 (54.5 %)	–
		**S**	10 (45.5 %)	–
Carbapenemase producers (*n* = 36)	**10^5^ CFU/mL**	**R**	36 (100 %)	–
		**S**	0	–
	**10^4^ CFU/mL**	**R**	29 (80.6 %)	–
		**S**	7 (19.4 %)	–
Total (*n* = 112)	**10^5^ CFU/mL**	**R**	85 (87.6 %)	0
		**S**	12 (12.4 %)	15 (100 %)
	**10^4^ CFU/mL**	**R**	55 (56.7 %)	0
		**S**	42 (43.3 %)	15 (100 %)

^*a*^ Values highlighted in gray correspond to the rate of very major errors, defined as a susceptible response by the Rapid Amp NP test obtained with strains expressing β-lactam resistance phenotypes.

^*b*^ No detection.

The MRSA strains tested did not give a positive signal using the Rapid Amp NP test, even when testing an inoculum at 10^5^ CFU/mL, and despite the corresponding bacterial colonies recovered from solid medium giving a positive result when tested with the nitrocefin disc test. It has been previously described that nitrocefin had unsatisfactory sensitivity for the detection of β-lactam resistant *S. aureus* (27, 28).

It is likely that the lack of detection of β-lactamase activity in many samples was actually related to a limited bacterial load in the tested samples, as supported by the observation that this test was positive for the same samples when higher concentrations (10^6^ to 10^7^ CFU/mL) were analyzed (data not showed). Of note also is the non-optimal detection of the β-lactamase activity among isolates belonging to species known to produce intrinsic β-lactamases, such as *E. cloacae* and *K. pneumoniae*, which did not display a positive result with the Rapid Amp NP test although being positive for the cefinase test. This suggests that the activity or amount of the intrinsic activity of the β-lactamases or the amount of those β-lactamases produced at natural state (one copy of the gene in the chromosome) may be insufficient to be detected considering the detection threshold of the test.

For the detection of ESBL producers, namely those producing CTX-M, SHV, TEM, and VEB β-lactamases, a positive result was obtained for 100% (22/22) of the tested strains within 5 to 60 min, and 95.4% (21/22) were positive within 20 min.

For the detection of carbapenemase producers, a positive result was obtained for 100% (36/36) of the tested strains. It allowed the rapid detection of nitrocefin hydrolysis resulting from all types of carbapenemases produced, namely, IMI, IMP, KPC, NDM, OXA-48-like, and VIM, within 5 to 60 min. Hence, the detection of β-lactamase activity is optimal for all carbapenemase producers using the Rapid Amp NP test, regardless of the respective type or levels of carbapenemase activity. Therefore, in a context of increasing prevalence of ESBL or carbapenemase worldwide, the Rapid Amp NP test perfectly detected aminopenicillin resistance in strains producing such enzymes.

Finally, the Rapid Amp NP test remained negative when performed with the 15 non-β-lactamase-producers, indicating that the test does not give positive results in the absence of a β-lactam hydrolysis activity. It should be noted that β-lactam resistance mechanism other than β-lactamases cannot be detected using this test, as it relies on the hydrolysis of nitrocefin. Thus, combining this test with rapid identification methods could help the orientation of the diagnostic established thanks to the test, especially regarding the natural resistance profile of the pathogen, or the possibility of samples containing two or more bacterial species. In fact, direct gram staining displays satisfying results in urine samples with high bacterial count (29) and could prove useful in combination with the result of our test, and various methods such as mass spectrometry (30–32), PCR (33), or mass sequencing (34), all able to be implemented directly from urine samples, have been developed and have demonstrated their effectiveness in a short turnaround time, some of them being also capable of investigating antibiotic resistance (31, 34, 35). However, such methods often require materials that are not always available in routine diagnostic laboratories, and the volume of urine available in a sample may not be sufficient to run both the Rapid Amp NP test and another rapid diagnostic test. It should, however, be kept in mind that the ultimate goal is to implement the Rapid Amp NP test at the general practitioner, where those techniques are not available.

One limitation of this test is that its sensitivity relies on the bacterial load. In fact, although a positive result was obtained for 87.6% of the tested β-lactamase-producing bacteria at 10^5^ CFU/mL (85/97), this rate dropped to 56.7% when testing sample containing 10^4^ CFU/mL (Table 2). Furthermore, a positive result was obtained for only 12 tested β-lactam producers at 10^3^ CFU/mL (12.4%), and none were obtained at 10^2^ CFU/mL. Among *E. coli*, those numbers were 95.4% at 10^5^ CFU/mL (62/65), 63.1% at 10^4^ CFU/mL (41/65), and 13.8% at 10^3^ CFU/mL (9/65). Although a bacterial load at 10^5^ CFU/mL is considered to be the standard for the diagnostic of UTIs (24), lower colony counts can also be considered significant (36) and recommendations tend to consider a lower detection threshold (19). Therefore, it is possible that false-negative results may be observed, should this test be implemented in routine diagnostics.

Overall, results of this proof-of-concept development suggested that the test will allow the detection of resistance to aminopenicillin directly from the urines. However, several shortcomings may limit its direct translation in clinical settings so far. First, its rate of very major errors, defined as a susceptible response to the Rapid Amp NP test with strains expressing β-lactam resistance phenotypes, makes difficult to draw a reliable diagnostic from its results. Second, although its turnaround time is short compared to colony-based testing, it still requires a preliminary 90 minutes incubation step which makes it difficult to implement in routine point-of-care diagnostics in addition to the material required for its implementation (incubator, centrifuge). Further work will be necessary to evaluate the performance of this test with urines from patients with non-complicated UTIs, as different parameters such as the time between sampling and testing, the bacterial growth in urine compared to growth in LB, release of β-lactamase, or the addition of borate in the sample for storage purposes may influence the performance of the test. We hypothesize that dead bacteria release a significant amount of β-lactamases over time in the bladder, therefore possibly increasing the hydrolysis amount of nitrocefin and consequently lowering the detection threshold of the test. Interestingly, 92% of cefazolin-resistant β-lactamase-producers were detected as positive at 10^5^ CFU/ml (81/88), suggesting that resistance to narrow-spectrum cephalosporin such as the orally given cefazolin is consistently detected. Cefazolin is used as a predictor of cephalosporin resistance in UTI caused by Enterobacterales (37); therefore, this test could also be an indicator for the efficacy of cephalosporins, which are mainly used in pediatric uncomplicated UTI. However, *Enterococcus* species naturally display low sensitivity to cephalosporins due to the production of penicillin-binding proteins PBP2b and PBP5 with low affinity to cephalosporins (38). Consequently, such resistance profiles will not be detected with the Rapid Amp NP test, as shown in Table 1.

To conclude, although several issues still need to be addressed, simplicity and rapid turnaround time to get result of the Rapid Amp NP test suggest that after further improvements, its use may be foreseen in medical laboratories and ultimately directly at the general practitioner from urines of the patients. Considering that the empirical treatment of UTI classically involves the use of broad-spectrum antibiotics such as fosfomycin, nitrofurantoin, ciprofloxacin, trimethoprim-sulfamethoxazole, its use may contribute to reconsidering the use of aminopenicillins (and even narrow-spectrum cephalosporins) as a first-line treatment for non-complicated UTI. In a world where antibiotic resistance is claimed as an urgent threat, this test may contribute to reducing the pression on emerging resistance worldwide.
